# Relationship between sodium and diffusion MRI metrics in multiple sclerosis

**DOI:** 10.1093/braincomms/fcae446

**Published:** 2025-01-06

**Authors:** Emilio Cipriano, Giacomo Boffa, Nicole Graziano, Claire Wigley, Maria Petracca, Simona Schiavi, Giovanni Luigi Mancardi, Sylvia Klineova, Fernando Boada, Fred Lublin, Matilde Inglese, Lazar Fleysher

**Affiliations:** Department of Neuroscience, Rehabilitation, Ophthalmology, Genetics, Maternal and Child Health (DINOGMI), University of Genoa, 16132 Genoa, Italy; Department of Neuroscience, Rehabilitation, Ophthalmology, Genetics, Maternal and Child Health (DINOGMI), University of Genoa, 16132 Genoa, Italy; Department of Neurology, IRCCS Ospedale Policlinico San Martino, 16132 Genoa, Italy; The Corinne Goldsmith Dickinson Center for Multiple Sclerosis, Icahn School of Medicine at Mount Sinai, New York, NY 10029, USA; The Corinne Goldsmith Dickinson Center for Multiple Sclerosis, Icahn School of Medicine at Mount Sinai, New York, NY 10029, USA; Department of Human Neurosciences, Sapienza University of Rome, 00185 Rome, Italy; Department of Neuroscience, Rehabilitation, Ophthalmology, Genetics, Maternal and Child Health (DINOGMI), University of Genoa, 16132 Genoa, Italy; Department of Neuroscience, Rehabilitation, Ophthalmology, Genetics, Maternal and Child Health (DINOGMI), University of Genoa, 16132 Genoa, Italy; The Corinne Goldsmith Dickinson Center for Multiple Sclerosis, Icahn School of Medicine at Mount Sinai, New York, NY 10029, USA; Radiological Sciences Laboratory, School of Medicine, Stanford University, Stanford, CA 94305, USA; The Corinne Goldsmith Dickinson Center for Multiple Sclerosis, Icahn School of Medicine at Mount Sinai, New York, NY 10029, USA; Department of Neuroscience, Rehabilitation, Ophthalmology, Genetics, Maternal and Child Health (DINOGMI), University of Genoa, 16132 Genoa, Italy; Department of Neurology, IRCCS Ospedale Policlinico San Martino, 16132 Genoa, Italy; Department of Radiology, Icahn School of Medicine at Mount Sinai, New York, NY 10029, USA

**Keywords:** multiple sclerosis, sodium MRI, intra-cellular and extra-cellular sodium concentration, diffusion MRI

## Abstract

Sodium MRI can measure *in vivo* sodium concentrations in people with multiple sclerosis, but the extent to which these alterations reflect metabolic dysfunction in the absence of tissue damage or neuroaxonal loss remains uncertain. Increases in total sodium concentration and extracellular sodium concentration are believed to be indicative of tissue disruption and extracellular space expansion. Conversely, increase in intracellular sodium concentration may represent early and transient responses to neuronal insult, preceding overt tissue damage. Here, we explored the relationship between total sodium concentration, intracellular sodium concentration and intracellular sodium volume fraction, which reflects extracellular sodium concentration, against histology-validated microstructural metrics obtained using diffusion MRI. Fifty-two individuals with multiple sclerosis and 26 healthy controls underwent ^1^H/^23^Na MRI. Microstructural parameters were derived using Diffusion Basis Spectrum Imaging and Neurite Orientation Dispersion and Density-Imaging models. A progressive worsening in total sodium concentration and intracellular sodium volume fraction was observed from HCs white matter to normal-appearing white matter and further into T2-hyperintense and T1-hypointense lesions. Both total sodium concentration and intracellular sodium volume fraction, but not intracellular sodium concentration, correlated with Neurite Orientation Dispersion and Density Imaging and Diffusion Basis Spectrum Imaging metrics within multiple sclerosis lesions. Our findings confirm the utility of total sodium concentration and extracellular sodium concentration as indicators of extracellular expansion and axonal loss and underscore intracellular sodium concentration as a valuable biomarker for metabolic dysfunction in multiple sclerosis.

## Teaser

Using sodium MRI, Cipriano *et al*. have shown that extracellular sodium concentration, but not intracellular sodium concentration, is associated with tissue damage and neuroaxonal loss in the brain of individuals with multiple sclerosis. Sodium MRI might help understanding *in vivo* multiple sclerosis pathology by detecting changes that precede visible damage.

## Graphical Abstract

**Graphical Abstract:**
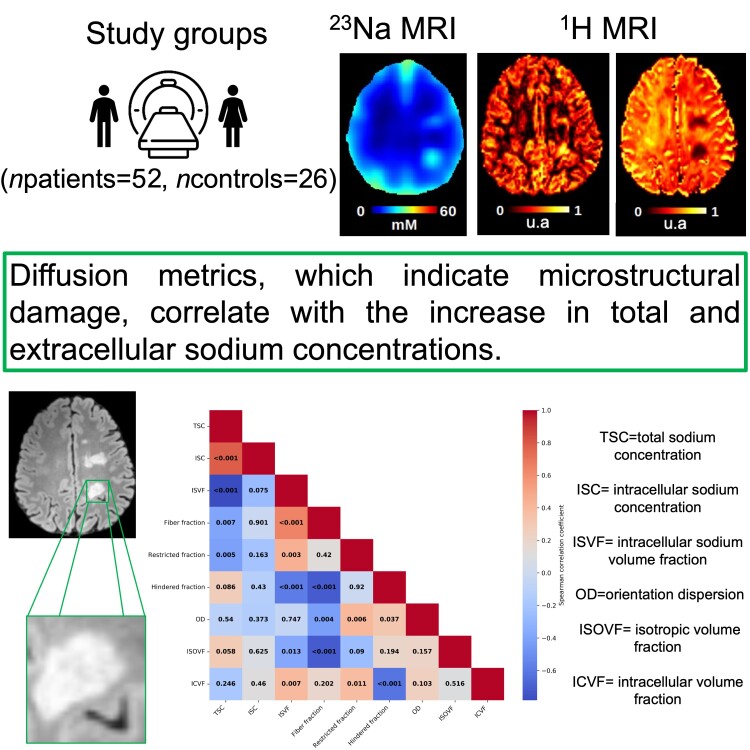

## Introduction

One potential mechanism contributing to neuroaxonal degeneration in multiple sclerosis (MS) is the dysregulation of sodium (Na+) homeostasis.^1^ In MS, sodium balance is disrupted by several factors affecting sodium influx, energy demand and the sodium gradient^2-4^. Following demyelination, a redistribution of voltage-gated Na+ channels along axons leads to an increased demand for ATP to maintain ion transmembrane concentration gradients. When this demand overwhelms Na+/K+ATPase pump function, a sustained Na+ influx results, increasing the intracellular sodium concentration (ISC). Elevated ISC triggers a progressive rise in intracellular Ca+ concentration, ultimately disrupting the axon-myelin unit, expanding the extracellular space, and increasing extracellular sodium concentration (ESC). Furthermore, acute inflammatory oedema may exacerbate ESC, contributing to axonal swelling in both lesions and normal-appearing white matter (NAWM).^5,6^

Sodium concentration in brain tissues can be measured *in vivo* using sodium MRI. Total sodium concentration (TSC), a weighted mean of intracellular (10–15 mM) and extracellular (∼140 mM) sodium concentrations, is elevated in MS lesions compared to the white matter of healthy controls and is associated with physical disability and cognitive impairment.^7^ This suggests that an increase in sodium is linked to neuronal loss and tissue disruption.

However, it remains unclear to what extent brain sodium concentrations detected by MRI reflect *in vivo* metabolic alterations in the absence of tissue damage or neuroaxonal loss and extracellular space expansion. An increase in TSC and ESC likely indicates tissue disruption and extracellular space expansion. On the contrary, an increase in ISC may be an early and transient phenomenon following neuronal insult, preceding overt tissue damage. Studies have shown that TSC increase correlates with reduced fractional anisotropy in the cervical spinal cord of MS patients^8^ and that increases in TSC in acute versus chronic MS lesions are associated with higher inversion recovery signal in acute lesions, indicating that neuroaxonal loss is a principal driver of TSC increase.^9^ Furthermore, myelin loss may lead to altered quadrupolar splitting and longer relaxation times, increasing the apparent quantification of TSC.^10^ Conversely, our group has found that ISC does not correlate with measures of tissue destruction such as T1 lesion volume and normalized grey matter volume.^11,12^ Similarly, concurrent ionic and metabolic mapping at 3T has shown that TSC increase is spatially related to mitochondrial neuronal dysfunction, suggesting that sodium imaging could serve as a biomarker of energetic dysfunction in MS.^13^

Validating sodium imaging with post-mortem histology is challenging due to immediate alterations in channel and ion homeostasis after death. Therefore, *in vivo* validation of sodium imaging with post-mortem confirmed metrics sensitive to MS pathology is crucial to differentiate the contributions of tissue damage and metabolic dysfunction to sodium increase.

In this study, we aimed to investigate the relationship between TSC, ISC and intracellular sodium volume fraction (ISVF), reflecting ESC,^14^ with histology-validated metrics of microstructural properties obtained using diffusion MRI. We employed two diffusion models, Diffusion Basis Spectrum Imaging (DBSI) and Neurite Orientation Dispersion and Density Imaging (NODDI), which have been validated in tissue phantoms, animal models, autopsied human MS spinal cord and biopsied human brain tissue^15-18^ to be specific and sensitive to cellularity and axonal density within MS lesions. Specifically, DBSI metrics of ‘restricted isotropic fraction’ and ‘fiber fraction’ positively correlated with cellularity and axonal density, while the ‘hindered fraction’ was associated with the extra-axonal compartment.^15,19^ The NODDI metric of intracellular volume fraction (ICVF) positively correlated with neurite density.^20^

Our hypothesis was that TSC and ISVF, but not ISC, would be associated with dMRI metrics of extracellular space expansion in brain tissues of individuals with MS.

## Materials and methods

### MRI imaging protocol

MRI examinations were performed on a Siemens Magnetom Prisma 3T with 64ch hydrogen and 8ch dual-tuned ^1^H/^23^Na head coils (Rapid Biomedical). The ^1^H imaging protocol consisted in: a 3D-MPRAGE (TR = 2400 ms, TE = 2.12 ms, TI = 1060 ms, voxel size = 1 × 1 × 1 mm^3^); 3D-FLAIR (TR = 5000 ms, TE = 393 ms, TI = 1800 ms, voxel size = 0.4 × 0.4 × 1.0 mm^3^); EPI spin echo sequence for multi-shell dMRI with 99 encoding directions scheme and maximum b-value = 2000s/mm^2^ (TR = 4700 ms, TE = 100 ms, voxel size = 2 ×2 × 2 mm^3^); an additional pair of spin echo EPI images (TR =8000 ms, TE = 66 ms, voxel size = 2 × 2 × 2 mm^3^) with opposite phase encoding directions was acquired for the susceptibility distortion correction of the dMRI images.

The ^23^Na acquisition protocol included a 3D-MPRAGE (TR = 2220 ms, TE = 3.3 ms, TI = 1100 ms, voxel size = 1 × 1 × 1mm^3^). The single-quantum (SQ) ^23^Na MRI was acquired using a Twisted Projection Imaging (TPI) sequence (TR = 100 ms, TE = 0.5 ms, voxel size = 3.43 × 3.43 × 3.43 mm^3^). The TPI sequence was also employed for triple-quantum filtered (TQF) ^23^Na MRI (TR = 100 ms, TE = 5 ms, voxel size = 3.43 × 3.43 × 3.43 mm^3^). The SQ and TQF ^23^Na MRI acquisitions were preceded by a manual B_0_ shim using the ^1^H MRI-based B_0_-shimming routine.

### Sodium MRI processing

The ^23^Na maps were generated using the canonical model of intracellular and extracellular sodium distribution.^21^ Our sodium MRI acquisition was based on the combination of SQ and TQF signals that depend on relaxation rates and flip angle.^14^ The SQ signal relies on a mono-exponential T_2_ decay and is used to quantify the TSC without separating the contribution of intra and extra sodium content.^22^ However, in the brain’s white and grey matter, the interaction between the electrical quadrupole moment of sodium and the electrical field gradients produced by the electrons of the molecular environment leads to the creation of a bi-exponential T_2_ decay signal, detectable using TQF sequences.^10^ Consequently, the TQF signal is used to interpret the intracellular sodium compartment.^14^ After acquisition, the contributions of intra and extra compartments were extracted. The TSC maps were derived from the total sodium signal using a linear method,^22^ with the vitreous humor of the eyes (TSC≈140 mM) and background noise (TSC≈0 mM) as reference concentrations.^23^ ISC and ISVF maps were obtained according to the method described by Fleysher *et al.*^14^ The partial volume effect (PVE) was reduced by subtracting the segmented maps of cerebrospinal fluid from each of the ^23^Na maps.^12^

### Structural MRI processing

T2-hyperintense and T1-hypointense lesion volumes were segmented on 3D-FLAIR and T1-weighted images using Jim version 7.0 (Xinapse Systems Ltd, UK). The T1-weighted images were then filled using FSL.^24^ The NAWM was obtained by subtracting FLAIR-hyperintense lesions from the segmented WM mask derived from the T1 filled images using FreeSurfer.^25^ The T2 and T1 lesion masks, along with NAWM, were registered with a nearest-neighbor interpolation on ^23^Na maps using ANTs.^26^ For accurate registration, the T1-weighted volume was affine-transformed to the 3D-MPRAGE obtained with the ^1^H/^23^Na, previously rigidly registered to the TSC map. To ensure reliable sodium quantification, which is dependent upon lesion volume,^27^ only lesions with a volume ≥0.41 cm^3^ (≥10 voxels at sodium resolution) were included, aiming to minimize PVE. Additionally, NAWM masks were eroded by one voxel to further reduce PVE.

### Diffusion MRI processing

dMRI images were first denoised using the Marchenko–Pastur principal component analysis algorithm in MRtrix3.^28^ Movement artefacts and susceptibility-induced distortions were corrected using *eddy* and *top-up* commands from FSL.^24^ Finally, we performed B_1_-field inhomogeneity correction via ANTs N4 algorithm.^29^

DBSI models dMRI signals to compute fibre fraction, hindered fraction and restricted fraction.^18^ These maps were obtained using a tool developed with Matlab (MathWorks).^30^

The NODDI model characterizes brain tissue yielding metrics such as orientation dispersion index, isotropic volume fraction (ISOVF) and ICVF.^20^ These maps were obtained using the AMICO software.^31^

Mean dMRI metrics values were extracted from WM lesions and NAWM, which were registered to the diffusion space through rigid transformation and nearest-neighbor interpolation using ANTs.

**Figure 1:**
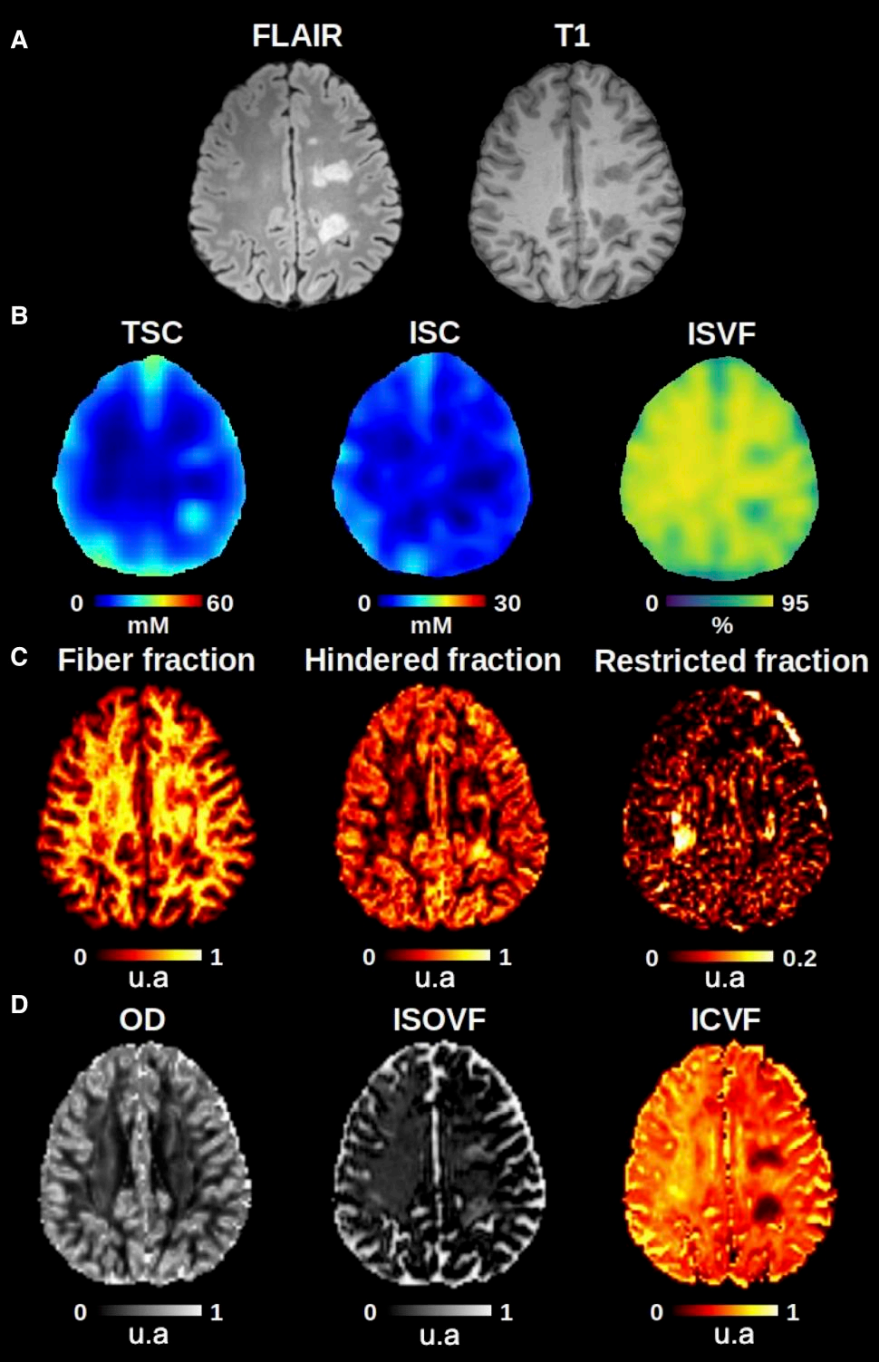
**Examples of the MRI sequences and metrics analysed in this study**. (**A**) Shows the T1-weighted and FLAIR images used to identify MS lesion. (**B**) The ^23^Na extracted maps are represented, including TSC, ISC and ISVF. (**C** and **D)** Show the DBSI maps (fibre fraction, hindered fraction and restricted fraction) and NODDI maps (OD, ISOVF and ICVF), respectively. In the selected images, 2 examples of MS lesions ≥0.41 cm^3^ are shown. FLAIR = fluid attenuated inversion recovery; TSC = total sodium concentration; ISC = intracellular sodium concentration; ISVF = intracellular sodium volume fraction; OD = orientation dispersion; ISOVF = isotropic volume fraction; ICVF = intracellular volume fraction.

Figure 1 reports the different sequences and metrics analysed in this study.

### Voxel-based analysis

The voxel-based analysis of ^23^Na and dMRI maps was conducted using the threshold-free cluster enhancement method (part of FSL, number of permutation = 5000).^32^ These maps were registered to the MNI space with affine and non-linear transformations using ANTs. Additionally, the T2 lesion masks were registered to MNI space to use them as confound regressors in the analysis.

### Statistical analysis

Statistical analyses were performed using Python (v.3.8.13). Two-sided *P*-values < 0.05 were considered significant. ANCOVA adjusted for age and sex was used to analyse differences in ^23^Na metrics and dMRI between the WM of HCs and NAWM/lesions of PwMS. The correlation between sodium metrics and dMRI in MS lesions as well as between sodium metrics, brain volumes and Expanded-Disability-Status-Scale (EDSS) scores were analysed using the Spearman correlation coefficient. Voxel-based statistical mapping analysis was used to assess differences in sodium concentration and dMRI between PwMS and HCs using the spatially normalized ^23^Na maps. Age, sex and lesion mask were used into this analysis as confound regressions (*P* < 0.05 corrected for family-wise error). The JHU White-Matter Tractography Atlas^33^ was utilized to identify the brain regions corresponding to the clusters that showed statistical differences between the groups.

## Results

### Study population

We prospectively enrolled 58 people with relapsing-remitting MS (PwMS) and 32 HCs. Six PwMS and six HCs were subsequently excluded from the analysis due to low quality of the MRI scans. A total of 52 PwMS [mean age (SD) of 36 ± 9 years] and 26 HCs (38 ± 13 years) were included in the final analyses. In PwMS, median EDSS was 2.0 (interquartile range = 1.0–3.0) and mean disease duration was 4.9 (±4.4) years. Table 1 reports main demographic, disease-related and MRI characteristics of the study groups. A mean number of 7.5 (SD = 5.5) lesions per subject was analysed.

**Table 1:** Demographics and MRI characteristics in patients with MS compared to HCs

	PwMS	HCs	*P*-value^a^
Age, years (mean, SD)	36.2 (9.33)	37.8 (13.1)	0.8
Female, number (%)	37 (71)	17 (65)	0.6
EDSS, median (IQR)	2.0 (1.0–3.0)		
Disease duration (mean, SD)	4.86 (4.42)		
Brain volume, ml (mean, SD)	1147 (127)	1154 (113)	0.9
T2 lesion volume, ml (mean, SD)	15.7 (17.6)		
T1 lesion volume, ml (mean, SD)	12.4 (14.2)		

IQR = interquartile range; PwMS = patients with multiple sclerosis; SD = standard deviation. a Mann–Whitney U-test and χ^2^ as appropriate.

### Sodium concentrations and dMRI-derived metrics in different brain tissues

**Figure 2:**
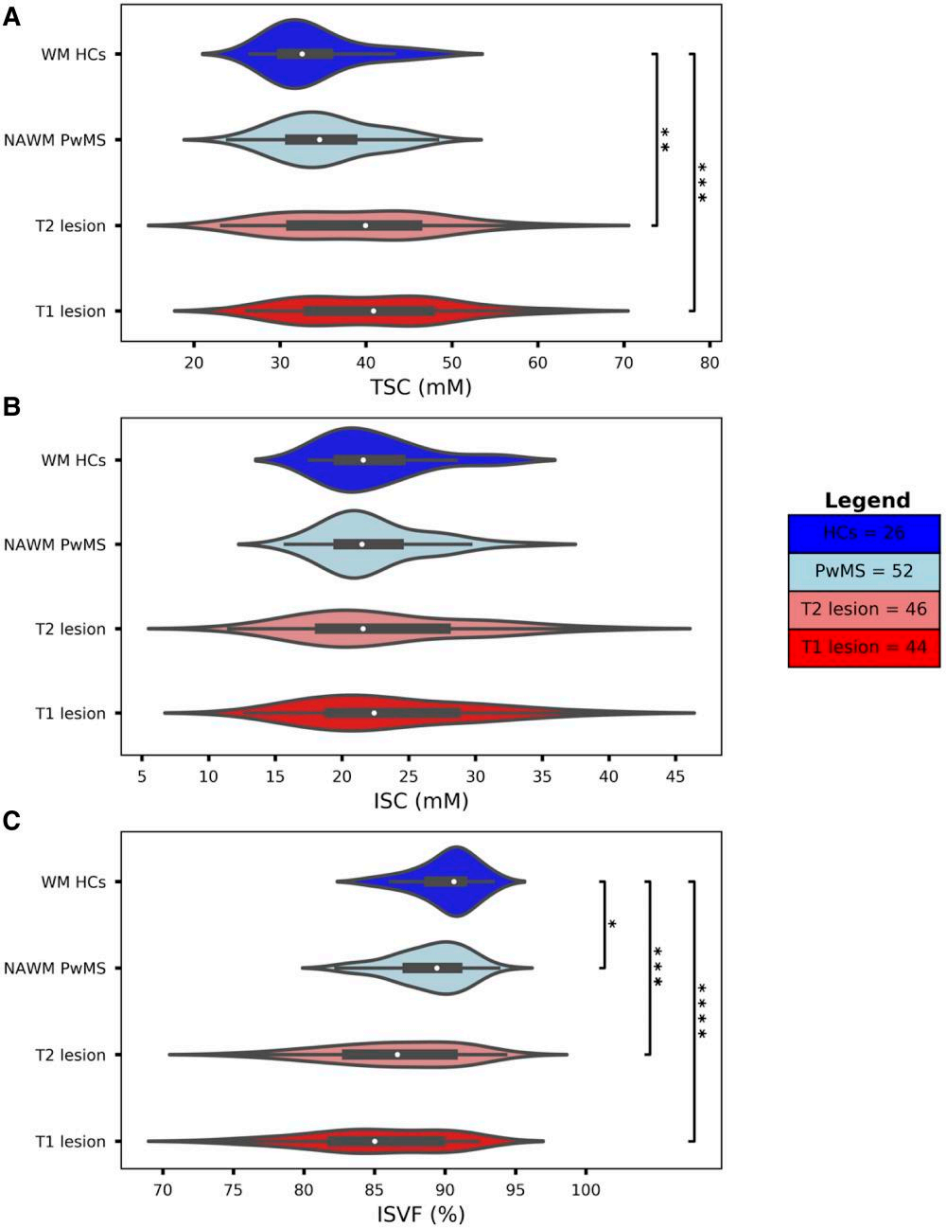
**Sodium concentration values for HCs and PwMS**. The violin plot combines the summary statistics of a box plot with the density estimation of the data distribution. The black box represents the interquartile range (25%–75% of the values), while the white points indicate the median of mean TSC **(A)**, ISC **(B)** and ISVF **(C)**. The width of the violin shows the kernel density estimation of the data. The asterisks indicate the significant differences obtained by the ANCOVA analysis (*P < 0.05, **P < 0.01, ***P < 0.001, ****P < 0.0001). The figure legend reports the sample size for each statistical test. HCs = healthy control subjects; PwMS = patients with multiple sclerosis; TSC = total sodium concentration; ISC = intracellular sodium concentration; ISVF = intracellular sodium volume fraction.

Figure 2 reports TSC, ISC and ISVF values in the WM of HCs, NAWM, T2-hyperintense lesions and T1-hypointense lesions. A worsening gradient WM->NAWM->T2-hyperintense lesions->T1-hypointense lesions in TSC was observed (33.9 mM->35.3 mM->39.1 mM->40.9 mM; *P* = 0.2, *P* < 0.01 and *P* < 0.001, respectively). A similar gradient was observed in ISVF (90%->89%->86%-> 85%; *P* < 0.05, *P* < 0.001 and *P* < 0.0001, respectively), while no differences were noted in ISC (22.4 mM->22.4 mM->23.2 mM->23.7 mM, all comparisons being not statistically significant).

A worsening gradient was observed in all dMRI metrics, with T1-hypointense lesions showing the most destructive values (Supplementary Figs 1 and 2).

### Associations between MS lesions sodium concentrations and dMRI-derived metrics

**Figure 3:**
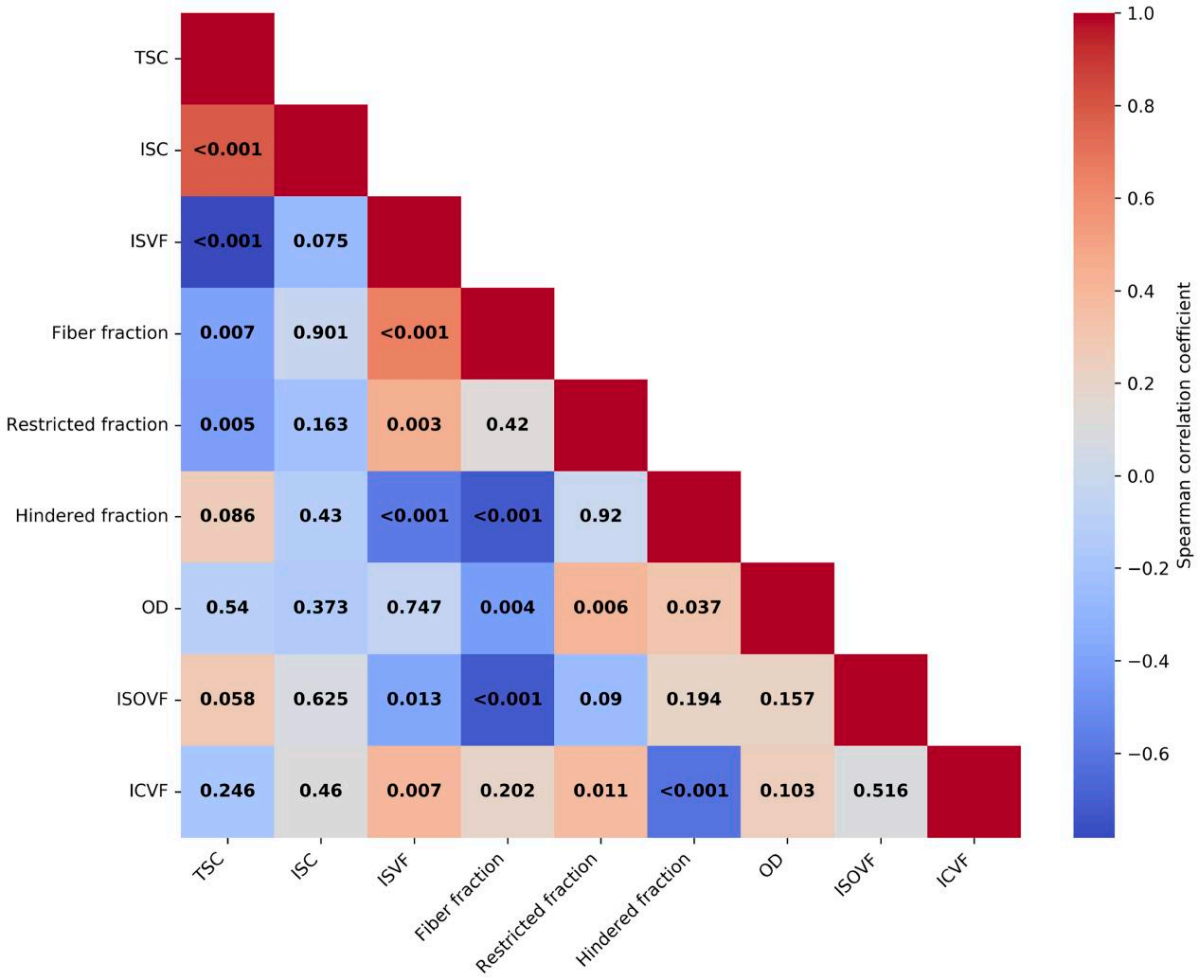
**Correlation matrix between sodium and dMRI metrics within T2-hyperintense MS lesions**. Spearman correlation coefficients were computed for sodium and dMRI metrics. The colour map represents the correlation coefficient, with the numerical value within each coloured box indicating the corresponding *P*-value. MS = multiple sclerosis, TSC = total sodium concentration; ISC = intracellular sodium concentration; ISVF = intracellular sodium volume fraction; OD = orientation dispersion; ISOVF = isotropic volume fraction; ICVF = intracellular volume fraction.

TSC and ISVF significantly correlated with most DBSI and NODDI parameters within T2-hyperintense MS lesions (Fig. 3), with the strongest associations found between fibre fraction and ISVF (*P* < 0.001, ρ = 0.65), ICVF and ISVF (*P* = 0.007, ρ=0.4) and restricted fraction and TSC (*P* = 0.005, ρ = −0.41). These results were also confirmed considering the T1-hypointense lesions (Supplementary Fig. 3). Conversely, ISC did not correlate with any of the DBSI or NODDI metrics.

### Sodium concentration and dMRI-derived metrics in NAWM

At voxel-wise analysis, we found diffuse areas of NAWM with significantly increased TSC (Table 2) and reduced ISVF (Table 3) in PwMS compared to HCs, encompassing several brain areas. Similar results were obtained at voxel-wise analyses using the DBSI and NODDI parameters (Supplementary Tables 1–3). No differences in ISC were noted between PwMS and HCs.

**Table 2:** Brain region of increased TSC in patients with MS compared to HCs

Cluster	Peak MNI coordinates (mm)			Cluster size	Brain regions
1	19	40	−8	906	Right inferior longitudinal fasciculus
2	−12	23	16	62	Forceps minor

Brain regions were identified according to the JHU White-Matter Tractography atlas. TFCE—*P* < 0.05 corrected—cluster size > 50 are shown. TFCE = threshold-free cluster enhancement.

**Table 3:** Brain region of decreased ISVF in patients with MS compared to HCs

Cluster	Peak MNI coordinates (mm)			Cluster size	Brain regions
1	50	−12	−25	1463	Right inferior longitudinal fasciculus
2	−36	−25	−18	670	Left inferior longitudinal fasciculus

Brain regions were identified according to the JHU White-Matter Tractography atlas. TFCE—*P* < 0.05 corrected—cluster size > 50 are shown. TFCE = threshold-free cluster enhancement.

TSC in the total NAWM was inversely correlated with restricted fraction (*P* = 0.019, ρ=−0.26), while ISVF was significantly correlated with fibre fraction (*P* = 0.002, ρ=0.22), restricted fraction (*P* = 0.008, ρ=0.49), hindered fraction (*P* = 0.001, ρ=−0.10) and ICVF (*P* = 0.047, ρ=0.27). No associations were noted between ISC and any of the DBSI or NODDI metrics in the NAWM. Similar results were obtained focusing on NAWM regions of significant difference in sodium-derived metrics between PwMS and HCs (Supplementary Figs 4 and 5).

**Figure 4:**
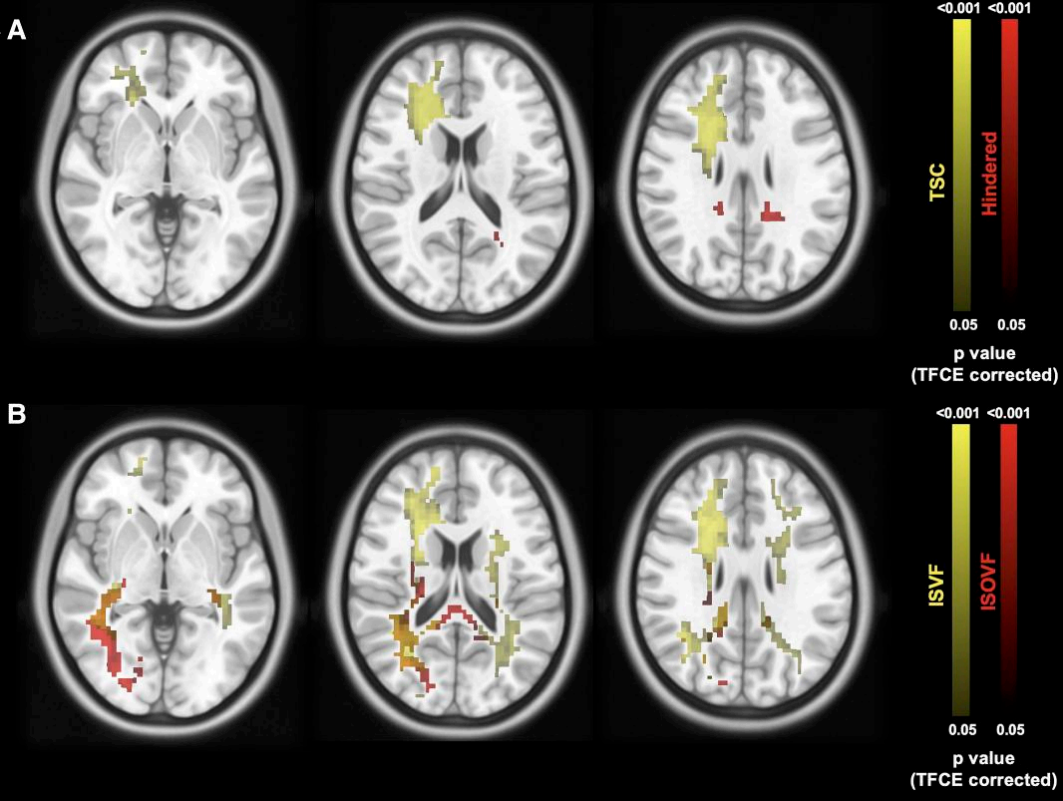
**Example of voxel-wise analysis of the sodium (yellow bar) and dMRI metrics (red bar)**. (**A**) The decrease in ISVF and the increase in ISOVF in the PwMS compared to HCs; (**B**) The increase in TSC and hindered fraction in the PwMS compared to HCs. The alteration maps are overlaid on a T1-weighted MNI template. The colour bar indicates the *P*-values (TFCE, *P* < 0.05 corrected for family-wise error). PwMS = patients with multiple sclerosis; TFCE = threshold-free cluster enhancement.

In order to compare the spatial overlap between clusters of altered TSC and ISVF and those of altered dMRI metrics, we overlaid the results of the previous analyses on the T1-weighted MNI template. Figure 4 illustrates some clusters of significantly altered sodium and dMRI metrics in PwMS compared with HCs. The spatial correspondence of abnormal sodium and dMRI metrics was minimal (dice scores ranging from 4% to 33%).

### Associations between sodium concentrations, brain volumes and clinical outcomes

We found that higher TSC and lower ISVF in the NAWM were associated with smaller total brain (*P* = 0.002, ρ=−0.42; *P* = 0.005, ρ=0.39, respectively), cortical (*P* = 0.005, ρ=−0.38; *P* = 0.029, ρ=0.30) and thalamic (*P* = 0.002, ρ=−0.42; *P* < 0.001, ρ=0.53) volumes. Higher TSC in T2-hyperintense and T1-hypointense lesions was also associated with smaller total brain volume (*P* = 0.035, ρ=−0.32; *P* = 0.047, ρ=−0.29, respectively), while lower ISVF within T2 and T1 lesions were associated with smaller thalamic volume (*P* = 0.001, ρ=0.39; *P* = 0.014, ρ=0.36). Higher ISC within NAWM was associated with smaller total brain and cortical volumes (*P* = 0.013, ρ=−0.34; *P* = 0.007, ρ=−0.37, respectively).

We observed a trend toward lower EDSS with higher ISVF in NAWM and T1-hypointense lesions (*P* = 0.06, ρ=−0.27 and *P* = 0.06, ρ=−0.29, respectively). The pyramidal functional sub score of EDSS was significantly associated with ISVF in T1-hypointense lesions (*P* = 0.03, ρ=−0.34). Additionally, the ambulation index was inversely correlated with ISVF in NAWM (*P* = 0.04, ρ=−0.29).

## Discussion

In this study, we investigated the *in vivo* relationships between TSC, ISC and ESC and their correlation with histology validated markers of microstructural damage obtained from diffusion MRI-based models. We confirmed that ESC, as estimated by the ISVF, is elevated in MS NAWM and lesions compared to WM of HCs. A worsening gradient was also found in dMRI metrics of axonal density and free water from HC WM to NAWM and MS lesions. Consistent with our hypothesis that an increase in ESC is indicative of neuroaxonal loss with expansion of the extracellular space, ESC was positively associated with dMRI metrics of free water and expansion of the extracellular compartment, and negatively associated with diffusion-derived metrics of axonal density. The interplay between sodium and dMRI metrics was confirmed in brain tissues affected by different degrees of MS-related damage (NAWM, T2-hyperintense and T1-hypointense focal lesions), corroborating the intrinsic association between elevated ESC, and consequently TSC, and dMRI markers of extracellular space expansion. Similarly, the associations between the increase in ESC and TSC with smaller brain volumes and worse clinical outcomes support the hypothesis that the two metrics detect neuronal loss and tissue disruption. On the other hand, in accordance with the hypothesis that ISC reflects metabolic abnormalities preceding structural damage, no associations were found between ISC and dMRI metrics of axonal density, cellularity, or increases in free water. These findings underscore the potential role of ISC as an early biomarker of metabolic dysfunction in MS, in the absence of overt tissue damage. Future studies incorporating myelin-sensitive metrics such as myelin water fraction, magnetization transfer ratio or g-ratio might further elucidate the role of ISC in assessing and monitoring tissue damage in MS.^34^

To investigate the spatial relationship between TSC, ESC and alterations in dMRI metrics, we performed a multimodal voxel-wise analysis of the NAWM. Interestingly, we identified several regions where altered sodium concentrations did not overlap with changes in dMRI metrics (and vice versa), suggesting that these two MRI sequences capture slightly different aspects of tissue disruption and extracellular expansion. We hypothesize that acute MS-related damage in the NAWM may be in some cases driven primarily by subtle inflammation, detectable by increased TSC without evident dMRI changes indicative of the disruption of the axonal-myelin unit. Conversely, in other circumstances such as incompletely remyelinated areas or in chronic inactive lesions, dMRI alterations without changes in sodium concentrations may indicate partial tissue repair in the absence of extracellular expansion. Although longitudinal studies are needed to test these hypotheses, the findings of this study suggest the potential for combined use of diffusion and sodium MRI.

One of the primary limitations of sodium imaging is its low spatial resolution. In the present study, we attempted to minimize the PVE by exclusively analysing MS lesions larger than 10 voxels at sodium resolution to obtain meaningful mean values for the three-sodium metrics. Although slightly higher ISC values were identified in lesions compared to PwMS NAWM and HC WM, no significant increase in ISC was observed in the brain tissues of people with MS. Previous findings described an ISC increase in the WM of PwMS^12^; however, that analysis did not focus on NAWM, and it is possible that those results were influenced by increased ISC in lesioned areas of patients. Moreover, ISC increases represent a transient and time-limited phase of metabolic dysfunction in the brain tissues of MS patients, suggesting that more specific patient populations might need to be studied, such as those early after a relapse or new lesion formation.

## Conclusion

Our findings support the roles of TSC and ESC as markers of extracellular disruption and axonal loss and highlight ISC as a promising biomarker of early metabolic dysfunction in MS.

## Supplementary Material

fcae446_Supplementary_Data

## Data Availability

Derived data supporting the findings of this study are available from the corresponding author on request.
