# Supplementary material for: Relationship between sodium and diffusion MRI metrics in multiple sclerosis
Source: Brain Commun. 2025 Jan 6;7(1):fcae446. doi: 10.1093/braincomms/fcae446 (PMC11702296; doi:10.1093/braincomms/fcae446)
Supplement: fcae446_Supplementary_Data [file fcae446_supplementary_data.docx]

# Supplementary materials


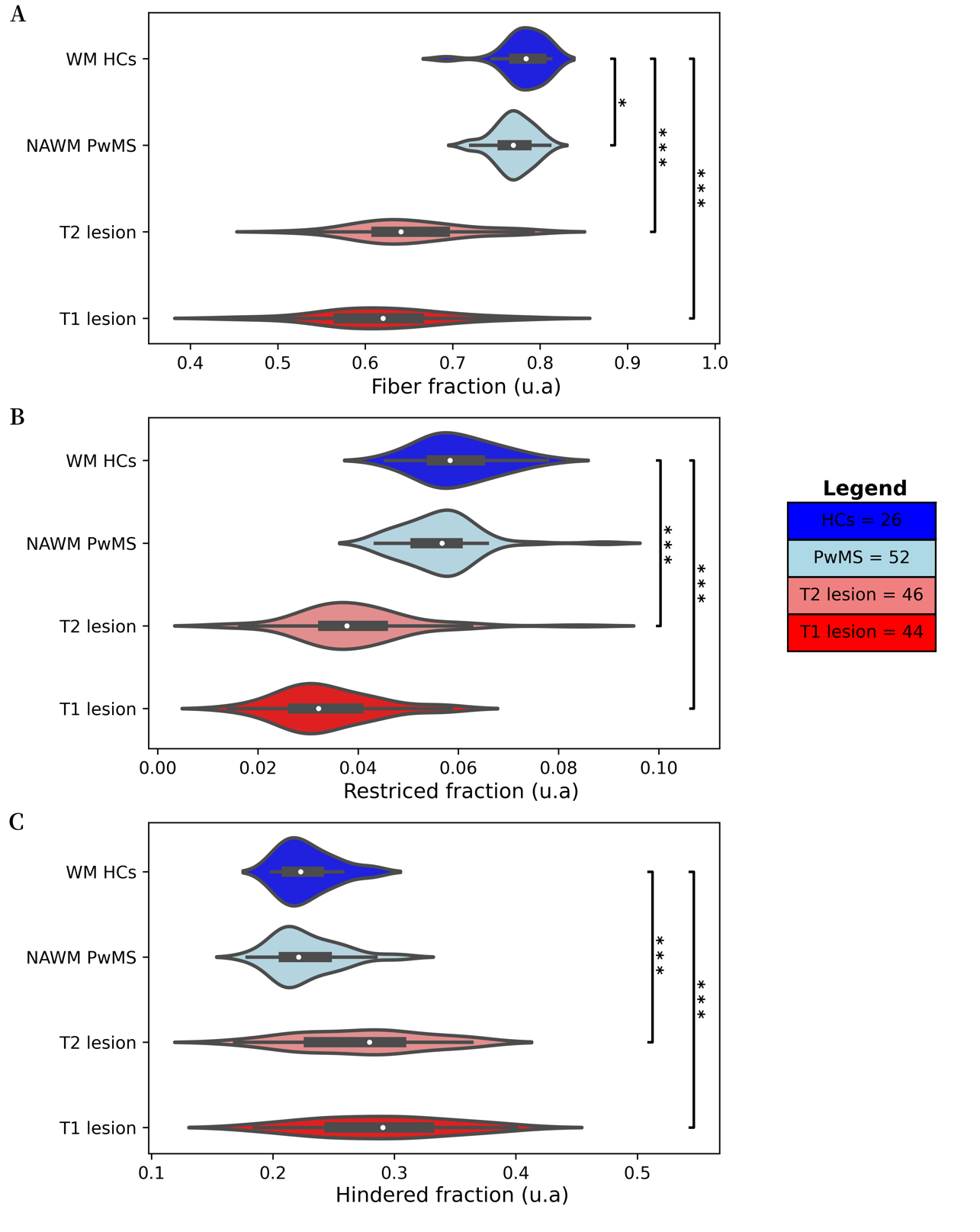


**Supplementary Figure 1: DBSI metrics for HCs and PwMS**. The violin plot combines the summary statistics of a box plot with the density estimation of the data distribution. The black box represents the interquartile range (25% to 75% of the values), while the white points indicate the median of mean Fiber fraction **(A),** Restricted fraction **(B)**, and Hindered fraction **(C)**. The width of the violin shows the kernel density estimation of the data. The asterisks indicate the significant differences obtained by the ANCOVA analysis (* p<0.05, ** p<0.01,*** p<0.001, **** p<0.0001). The figure legend reports the sample size for each statistical test. HCs = healthy control subjects; PwMS= patients with multiple sclerosis.


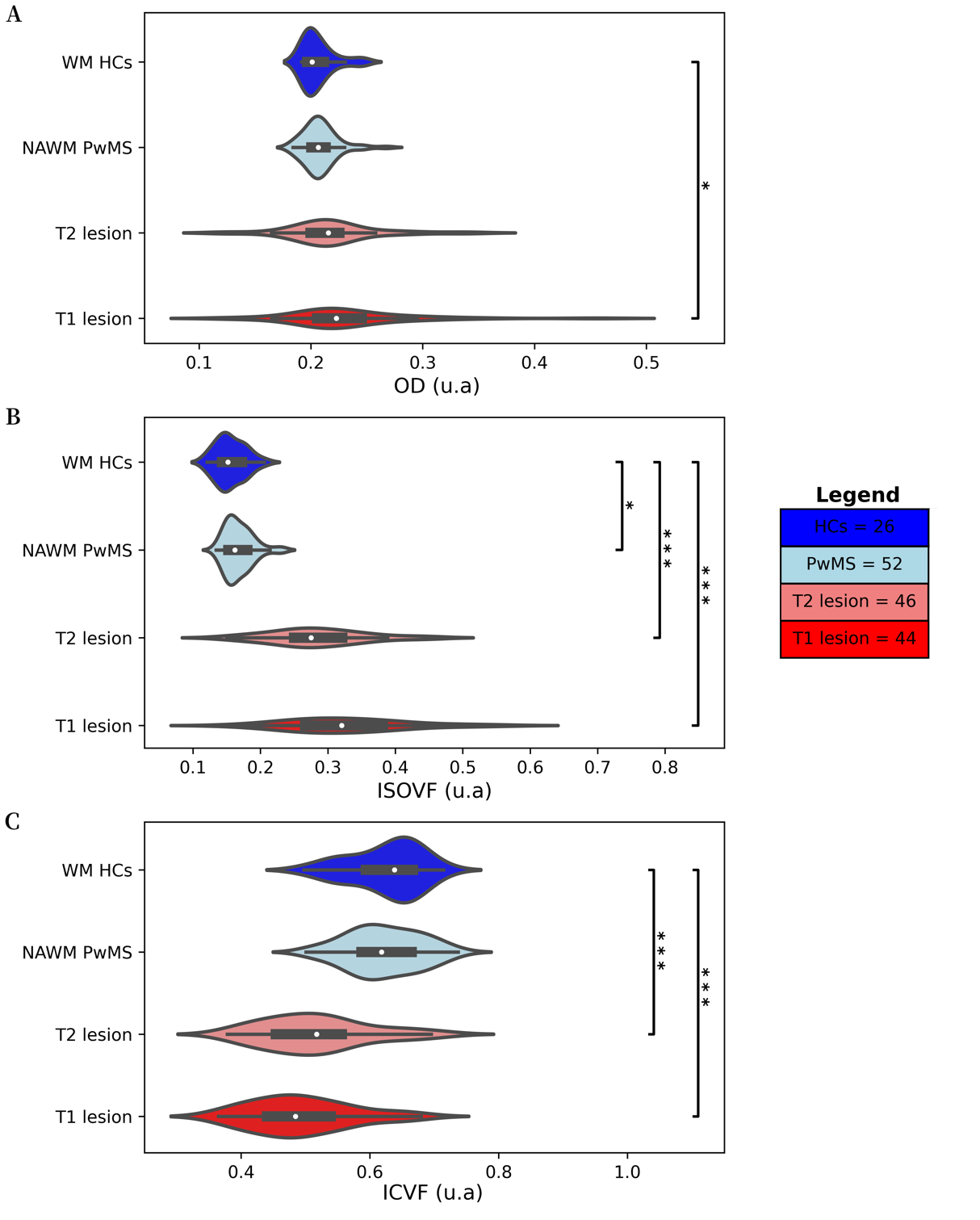


**Supplementary Figure 2: NODDI metrics for HCs and PwMS**. The violin plot combines the summary statistics of a box plot with the density estimation of the data distribution. The black box represents the interquartile range (25% to 75% of the values), while the white points indicate the median of mean OD **(A)**, ISOVF **(B)**, and ICVF **(C)**. The width of the violin shows the kernel density estimation of the data. The asterisks indicate the significant differences obtained by the ANCOVA analysis (* p<0.05, ** p<0.01,*** p<0.001, **** p<0.0001). The figure legend reports the sample size for each statistical test. HCs = healthy control subjects; PwMS= patients with multiple sclerosis; OD=orientation dispersion; ISOVF= isotropic volume fraction; ICVF= intracellular volume fraction.


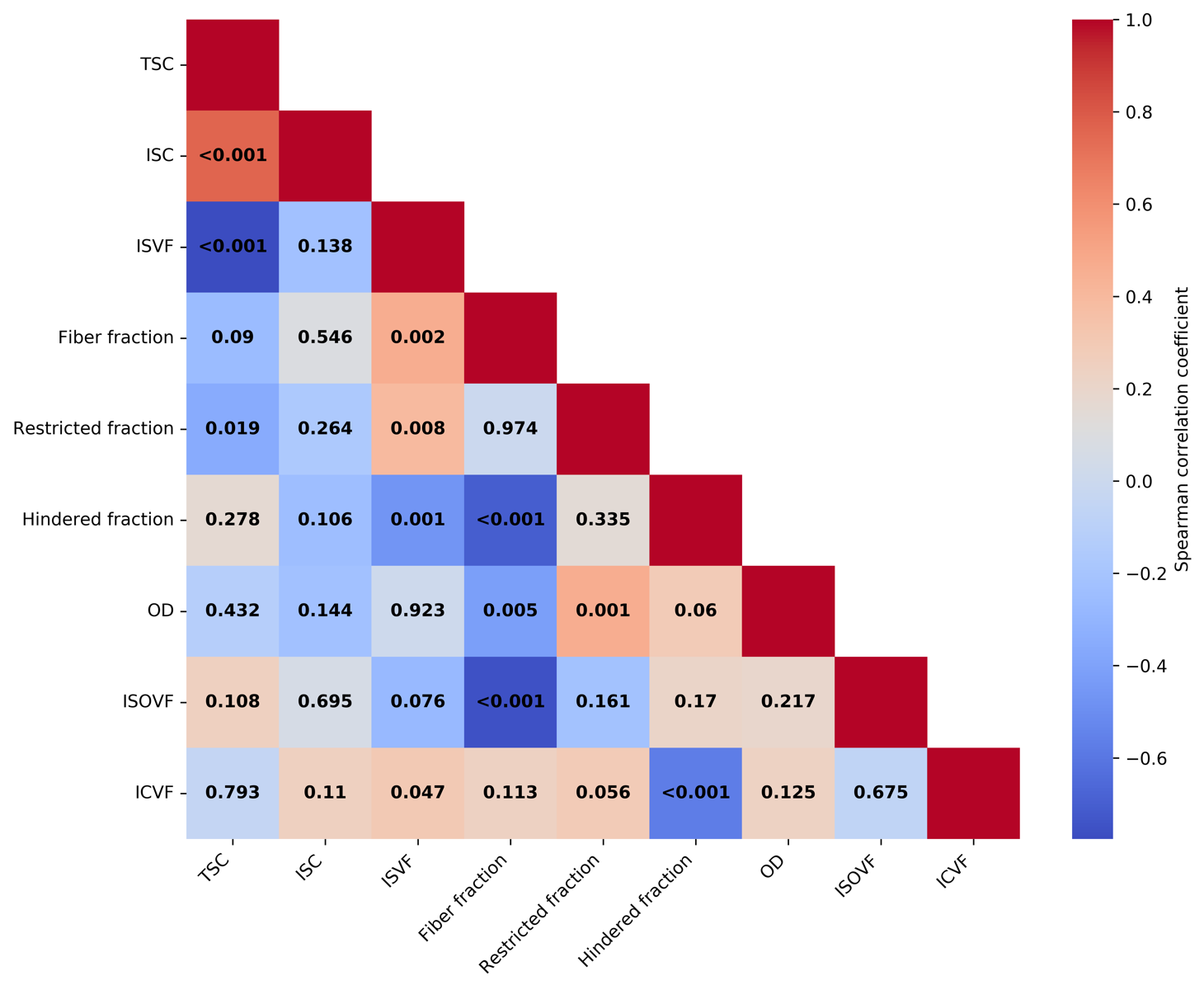


**Supplementary Figure 3**: **Correlation matrix between sodium and dMRI metrics within T1-hypointense MS lesions**. Spearman correlation coefficients were computed for sodium and dMRI metrics. The color map represents the correlation coefficient, with the numerical value within each colored box indicating the corresponding p-value. MS= multiple sclerosis; TSC=total sodium concentration; ISC= intracellular sodium concentration; ISVF= intracellular sodium volume fraction; OD=orientation dispersion; ISOVF= isotropic volume fraction; ICVF= intracellular volume fraction.

**Supplementary Table 1: Brain region of decreased hindered fraction in patients with multiple sclerosis compared to healthy controls**.

| **Cluster** | **peak MNI coordinates (mm)** | | | **Cluster size** | **Brain regions** |
| --- | --- | --- | --- | --- | --- |
| 1 | -12 | -45 | 23 | 63 | Splenium of corpus callosum |

Brain regions were identified according to the JHU White-Matter Tractography atlas.

TFCE – p<0.05 corrected – cluster size > 50 are shown.

TFCE = threshold-free cluster enhancement

**Supplementary Table 2: Brain region of increased OD in patients with multiple sclerosis compared to healthy controls**.

| **Cluster** | **peak MNI coordinates (mm)** | | | **Cluster size** | **Brain regions** |
| --- | --- | --- | --- | --- | --- |
| 1 | 50 | -28 | -14 | 402 | Right superior longitudinal fasciculus |

Brain regions were identified according to the JHU White-Matter Tractography atlas.

TFCE – p<0.05 corrected – cluster size > 50 are shown.

TFCE = threshold-free cluster enhancement

OD = orientation dispersion

**Supplementary Table 3: Brain region of increased ISOVF in patients with multiple sclerosis compared to healthy controls**.

| **Cluster** | **peak MNI coordinates (mm)** | | | **Cluster size** | **Brain regions** |
| --- | --- | --- | --- | --- | --- |
| 1 | 2 | -42 | -52 | 1632 | Brainstem |
| 2 | 53 | -15 | 23 | 51 | Right superior longitudinal fasciculus |

Brain regions were identified according to the JHU White-Matter Tractography atlas.

TFCE – p<0.05 corrected – cluster size > 50 are shown.

TFCE = threshold-free cluster enhancement

ISOVF = isotropic volume fraction

**
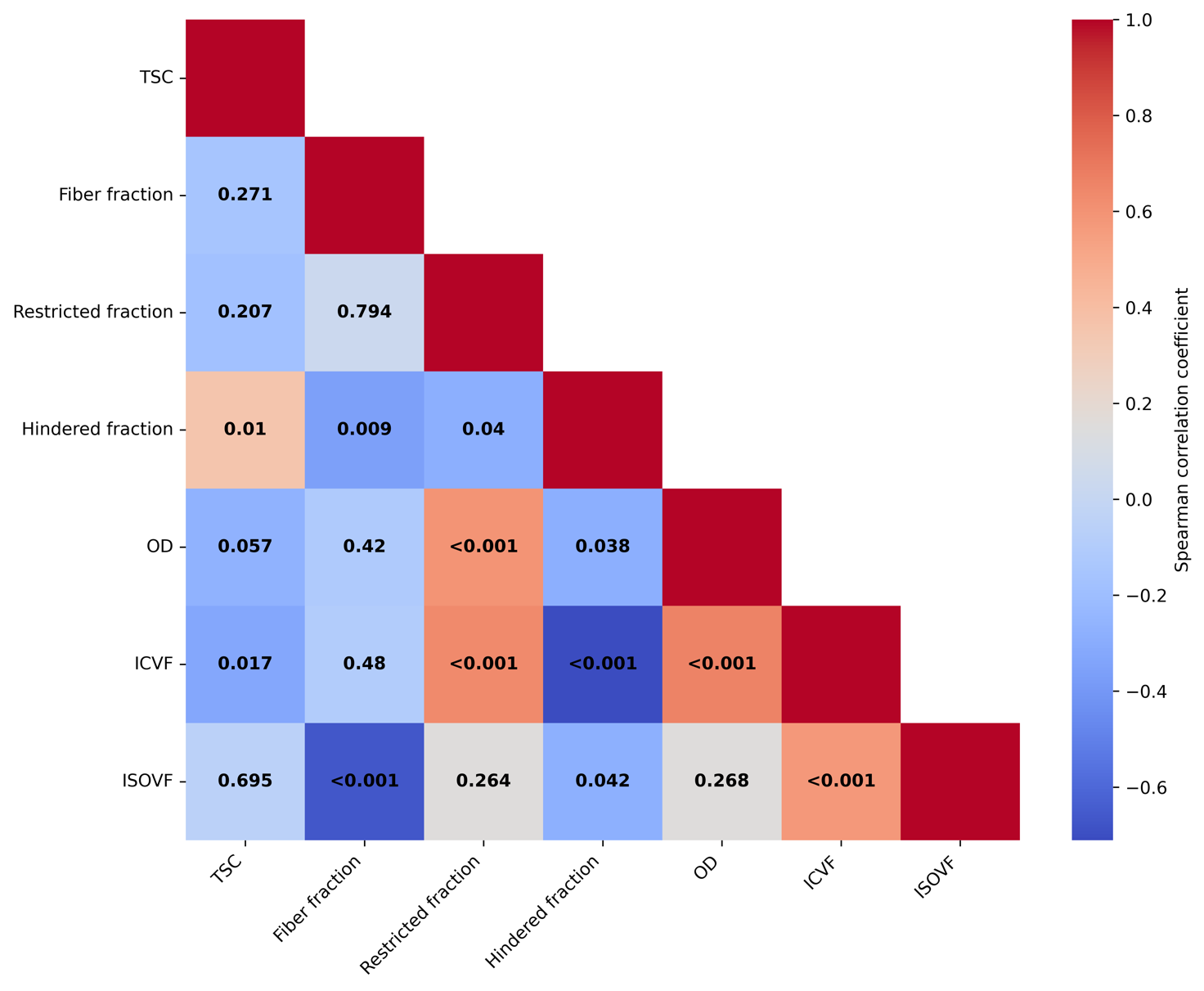
**

**Supplementary Figure 4**: **Correlation matrix between TSC and dMRI metrics within** **areas of altered TSC**. Spearman correlation coefficients were computed for TSC and dMRI metrics. The color map represents the correlation coefficient, with the numerical value within each colored box indicating the corresponding p-value. MS= multiple sclerosis; TSC=total sodium concentration; OD=orientation dispersion; ISOVF= isotropic volume fraction; ICVF= intracellular volume fraction.


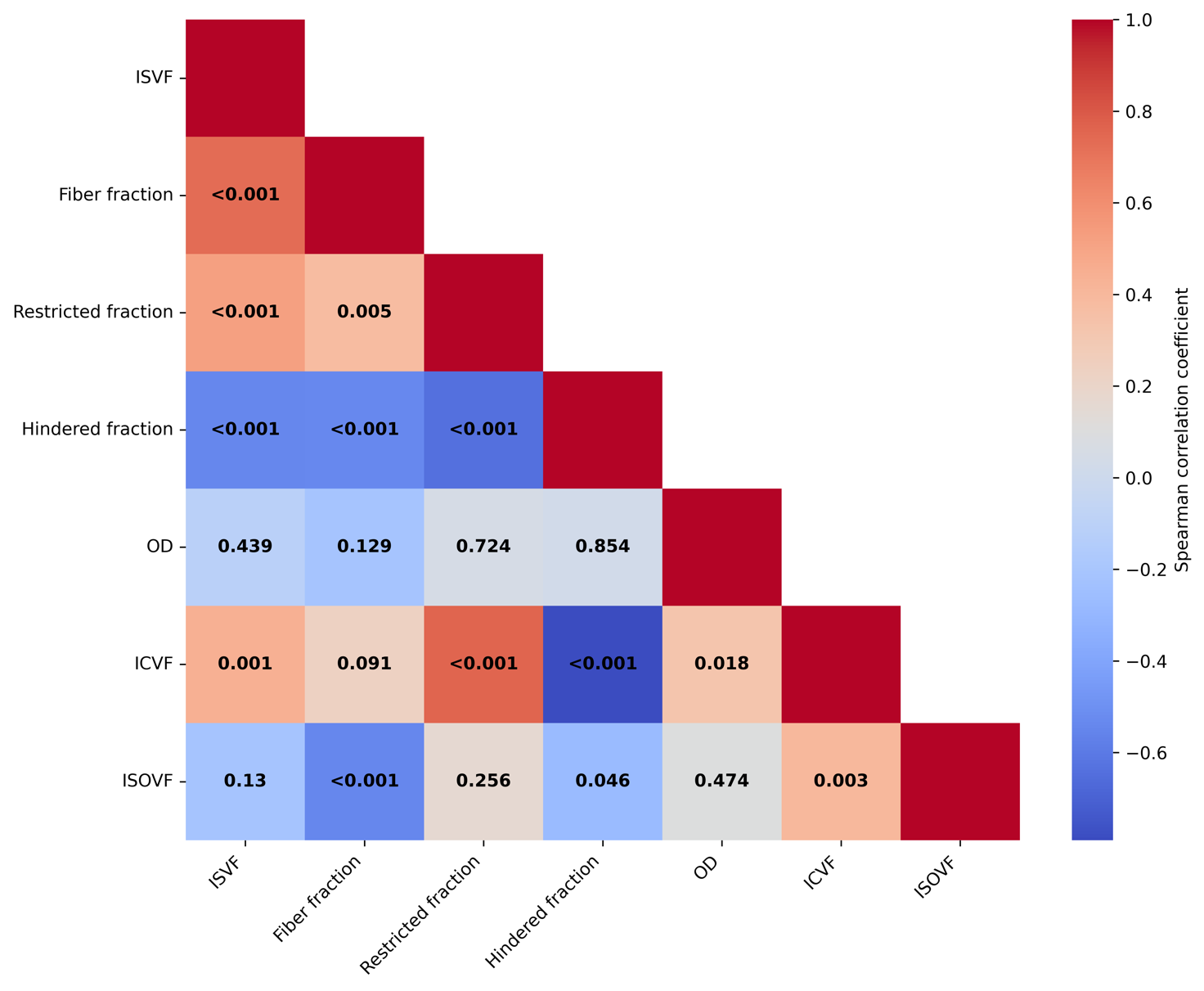


**Supplementary Figure 5**: **Correlation matrix between ISVF and dMRI metrics within areas of altered ISVF**. Spearman correlation coefficients were computed for TSC and dMRI metrics. The color map represents the correlation coefficient, with the numerical value within each colored box indicating the corresponding p-value. MS= multiple sclerosis; ISVF= intracellular sodium volume fraction; OD=orientation dispersion; ISOVF= isotropic volume fraction; ICVF= intracellular volume fraction.
